# Interobserver Agreement Between Artificial Intelligence, Radiologist, and Gynecologist in Hysterosalpingography Interpretation: A Retrospective Comparative Study

**DOI:** 10.3390/diagnostics16162576

**Published:** 2026-08-15

**Authors:** Deniz Taşkıran, Serdar Aslan, Salih Kolsuz, Mesut Alçı, Esra Yazgan Yiğitbaş

**Affiliations:** 1 Department of Obstetrics and Gynecolog, Faculty of Medicine, Giresun University, 28100 Giresun, Turkey; 2 Departments of Radiology, Faculty of Medicine, Giresun University, 28100 Giresun, Turkey; 3 Giresun Maternity and Children’s Hospital, Training and Research Hospital, 28100 Giresun, Turkey

**Keywords:** hysterosalpingography, infertility, artificial intelligence, interobserver agreement, tubal patency

## Abstract

**Background:** Infertility is a common reproductive health disorder that affects roughly 10–15% of couples during their reproductive period. Hysterosalpingography (HSG) is a widely utilized imaging modality for assessing uterine cavity morphology and fallopian tube patency and continues to play a central role in infertility investigations. Nevertheless, the interpretation of HSG findings may vary according to the experience and expertise of the evaluator, potentially leading to inconsistencies in clinical decision-making. Although artificial intelligence (AI) has demonstrated considerable potential in medical image analysis across various specialties, evidence regarding its application in the interpretation of HSG examinations remains scarce. Therefore, this study aimed to evaluate the level of agreement among radiologists, gynecologists, and an AI-based system in the assessment of identical HSG images. **Methods:** In this retrospective study, a total of 1443 HSG images obtained from 414 women who underwent hysterosalpingography as part of an infertility evaluation between January 2021 and January 2025 were reviewed. Cases with incomplete clinical records or suboptimal image quality were excluded from the analysis. All examinations were independently assessed by an experienced radiologist, a gynecologist specializing in infertility management, and a multimodal artificial intelligence system based on ChatGPT-5, with each evaluator blinded to the assessments of the others and to the patients’ clinical information. Image interpretation included the evaluation of contrast distribution, peritoneal spill, uterine cavity findings, tubal patency, and overall HSG impression, which were categorized according to predefined diagnostic criteria. The primary outcome was the degree of interobserver agreement among the evaluators. Agreement analyses were performed using Cohen’s kappa (κ) and Gwet’s AC1 coefficients. Analyses were conducted using IBM SPSS Statistics (version 30.0; IBM Corp., Armonk, NY, USA) and R statistical software (version 4.4.0; R Foundation for Statistical Computing, Vienna, Austria). Statistical significance was set at *p* < 0.05 (two-sided). **Results:** A total of 1443 HSG images obtained from 414 women were included in the final analysis. The mean age of the study population was 30.97 ± 5.59 years, and primary infertility accounted for 87.9% of cases. The average number of images acquired per examination was 3.49 ± 1.05. According to Cohen’s kappa analysis, the highest levels of agreement were observed for the assessment of image artifacts and contrast medium distribution. Agreement between the AI system and the radiologist was particularly strong for contrast medium distribution (κ = 0.757). For the overall interpretation of HSG findings, AI demonstrated substantial agreement with the radiologist (κ = 0.637), exceeding the level of agreement observed between the radiologist and the gynecologist (κ = 0.363). In contrast, concordance involving AI was lower for the evaluation of uterine abnormalities, intrauterine filling defects, and tubal patency. When agreement was reassessed using Gwet’s AC1 statistic, concordance coefficients were consistently higher than the corresponding kappa values across all evaluator pairs. Near-perfect agreement between AI and the radiologist was identified for contrast medium distribution (AC1 = 0.954), peritoneal spill (AC1 = 0.893), and patterns of peritoneal contrast passage (AC1 = 0.841). Procedures performed under local anesthesia yielded a significantly greater number of images than those conducted under general anesthesia (3.86 ± 0.86 vs. 3.08 ± 1.10, *p* < 0.001). No significant associations were detected between abnormal HSG findings and either infertility type or anesthetic technique. In multivariable analysis, the use of general anesthesia was independently associated with a lower image count, whereas the presence of tubal pathology emerged as an independent predictor of acquiring a greater number of images during the examination. **Conclusions:** Our findings indicate that AI-assisted interpretation of HSG images has the potential to complement expert assessment, showing substantial concordance in several key diagnostic domains. While the technology appears promising as a decision-support tool in infertility evaluation, further research and refinement are warranted, particularly regarding the assessment of tubal and uterine pathologies.

## 1. Introduction

Infertility affects approximately 10–15% of couples during their reproductive years and represents an important global health concern with medical, psychological, and socioeconomic consequences [1]. Evaluation of the uterine cavity and fallopian tube patency is an essential component of female infertility assessment. Hysterosalpingography (HSG) is widely used for this purpose, providing information on uterine anatomy and tubal patency through contrast-enhanced fluoroscopic imaging [1]. Owing to its accessibility, relatively low cost, and established role in assessing tubal patency, HSG remains an important component of routine infertility evaluation.

Although HSG provides valuable information about uterine and tubal anatomy, its interpretation may vary among observers. Evaluation of uterine anomalies, intrauterine filling defects, tubal obstruction, and peritubal adhesions often requires clinical expertise, and interobserver variability may influence diagnostic and therapeutic decision-making [2]. Radiologists generally focus on structural and morphological imaging findings, whereas gynecologists may interpret these findings in the context of infertility history, patient characteristics, and treatment planning. Consequently, the same HSG examination may be interpreted differently depending on the evaluator’s clinical perspective.

Artificial intelligence (AI) has become increasingly important in medical imaging. Convolutional neural network (CNN)-based deep learning models have shown promising results in various radiological image interpretation tasks and may support workflow efficiency and clinical decision-making [3,4]. AI-based systems have also demonstrated considerable potential across a range of radiological applications [5,6]. In obstetrics and gynecology, AI has been investigated for several diagnostic and prognostic applications, including the characterization of adnexal lesions, assessment of pelvic floor disorders, prediction of lymphatic dissemination in endometrial malignancies, and evaluation of fetal lung maturation [7,8,9]. In the field of infertility, accumulating evidence suggests that artificial intelligence holds substantial promise for enhancing the interpretation of imaging data and supporting clinical decision-making through advanced predictive and decision-support models [10]. More recently, large language models (LLMs) and multimodal AI systems have expanded the ability of AI to process different types of clinical information, including medical images. A large-scale review of 250 AI studies emphasized the importance of collaboration between healthcare professionals and computational researchers for the development of trustworthy and clinically applicable AI systems [11]. The economic impact of artificial intelligence in medical imaging is projected to grow substantially, accompanied by increasing integration of AI-driven technologies into routine clinical practice [12]. Furthermore, the incorporation of artificial intelligence into routine diagnostic workflows may alleviate the burden of time-consuming interpretative tasks, allowing specialists to focus on more complex clinical responsibilities and patient-centered decision-making [13]. In obstetric imaging, AI-assisted tools have demonstrated good-to-excellent reproducibility for selected fetal biometric measurements, although performance varies according to the anatomical structure evaluated [14].

Recent advances in large language models and multimodal artificial intelligence systems have extended the capabilities of AI beyond text-based applications, enabling the interpretation of visual and imaging data. Despite these developments, the role of AI in radiological image interpretation remains incompletely defined, and evidence regarding its application in specialized imaging modalities such as hysterosalpingography is still limited. To the best of our knowledge, no previous study has comprehensively compared the level of agreement among radiologists, gynecologists, and a multimodal AI system in the interpretation of HSG examinations. This study aimed to compare the interpretation of identical HSG images by radiologists, gynecologists, and a multimodal AI system and to assess the degree of interobserver agreement among them, thereby exploring the potential role of AI-assisted HSG interpretation.

## 2. Materials and Methods

### 2.1. Study Design and Population

This retrospective observational study included HSG examinations performed between January 2021 and January 2025 in women undergoing infertility evaluation at the Department of Obstetrics and Gynecology, Giresun Gynecology, Obstetrics and Pediatrics Training and Research Hospital. Retrospective data collection and evaluation were initiated only after approval was obtained from the local Ethics Committee (approval no. 2026/01).

A total of 1443 HSG images from 414 patients were included in the study. All available images from each HSG examination were reviewed, and the findings were integrated into a single examination-level assessment. Therefore, although 1443 individual images were evaluated, the unit of analysis for interobserver agreement was the HSG examination (n = 414). Cases with incomplete imaging records, insufficient image quality for reliable interpretation, or unavailable relevant clinical information were excluded.

Written informed consent, specifically including consent for AI-assisted evaluation of HSG images, was obtained from all participants. All HSG images were fully anonymized before being uploaded to the AI system, and no patient-identifying information or associated clinical data were provided to the model.

### 2.2. HSG Image Assessment

All examinations were independently reviewed by three evaluators: a radiologist and a gynecologist, each with at least four years of clinical experience and expertise in infertility imaging and HSG interpretation, and a multimodal artificial intelligence system based on ChatGPT-5. Each evaluator was blinded to the patients’ clinical characteristics, infertility history, and the assessments made by the other reviewers.

For every HSG study, findings were classified according to predefined categorical criteria. Specifically, contrast medium distribution was categorized as normal or abnormal; peritoneal spill as present or absent; image artifacts as present or absent; uterine cavity filling as adequate or inadequate; intrauterine filling defects as present or absent; uterine anomalies as present or absent; right and left tubal patency as patent or occluded; peritoneal spill pattern as normal or abnormal; and the overall HSG interpretation as normal or abnormal.

### 2.3. Artificial Intelligence Evaluation

AI-assisted image interpretation was performed using GPT-5 via the ChatGPT web interface (OpenAI, San Francisco, CA, USA) between February and March 2026. The model/version information available to the investigators through the user interface at the time of analysis was recorded as GPT-5; no specific model snapshot or build identifier was recorded.

HSG images were uploaded in PNG format at their available resolutions, which varied across examinations (e.g., 615 × 711, 760 × 587, and 814 × 672 pixels), with a 32-bit depth and 96–120 dpi. Images were evaluated without prior resolution standardization. All images were anonymized before analysis, and no patient-identifying information was provided to the AI system.

All HSG examinations were evaluated using the same predefined standardized prompt. For each patient, all images from the same HSG examination were presented sequentially within a single session, and the AI system was instructed to provide a unified assessment for the examination using predefined categorical response options. The standardized prompt was as follows:

“Evaluate the provided HSG image separately for each of the following parameters and report the result using the specified categorical options.

1. Contrast medium distribution: normal/abnormal
2. Peritoneal spill: present/absent
3. Image artifact: present/absent
4. Uterine cavity filling: adequate/inadequate
5. Uterine filling defect: present/absent
6. Uterine anomaly: present/absent
7. Right tubal patency: patent/occluded
8. Left tubal patency: patent/occluded
9. Peritoneal spill pattern: normal/abnormal
10. Overall HSG interpretation: normal/abnormal.”

The same prompt, image-presentation procedure, and categorical output format were used for all examinations. Each HSG examination was evaluated in a separate session, with the conversational context reset between patients, and response regeneration was permitted when necessary. No clinical information, demographic characteristics, infertility history, laboratory results, or previous imaging findings were provided to the AI system; therefore, the assessments were based solely on the submitted HSG images. The resulting categorical outputs were recorded and used directly in the interobserver agreement analyses.

The model was not trained, fine-tuned, or calibrated using the study dataset. Human reviewers and the AI system completed their assessments independently and were blinded to each other’s interpretations. AI-generated assessments were reviewed only for completeness and conformity with the predefined output format, without correction or modification of their content.

### 2.4. Study Outcomes

The primary endpoint was the degree of interobserver agreement among the radiologist, gynecologist, and AI system for HSG interpretation.

Secondary endpoints comprised agreement analyses for specific HSG findings and overall diagnostic impressions, identification of factors influencing the number of images obtained during HSG examinations, and evaluation of associations between infertility-related characteristics and abnormal imaging findings.

### 2.5. Statistical Analysis

Statistical analyses were carried out using IBM SPSS Statistics for Windows (version 30.0; IBM Corp., Armonk, NY, USA) and R statistical software (version 4.4.0; R Foundation for Statistical Computing, Vienna, Austria).

The normality of continuous variables was evaluated through the Shapiro–Wilk test in conjunction with histogram-based visual assessment. Variables showing a normal distribution were reported as mean ± standard deviation (SD), whereas non-normally distributed data were described using the median and range (minimum–maximum). Categorical variables were summarized as counts and percentages.

Between-group comparisons for continuous variables were performed using either the independent-samples *t*-test or the Mann–Whitney U test, depending on distributional characteristics. Differences in categorical variables were assessed using Pearson’s chi-square test or Fisher’s exact test when appropriate. Relationships between clinical characteristics and binary outcomes were investigated using univariable logistic regression models, and the findings were expressed as odds ratios (ORs) with corresponding 95% confidence intervals (CIs).

To determine factors independently associated with the number of HSG images acquired per patient, a multivariable linear regression model was constructed. The results were presented as regression coefficients (β) together with their 95% confidence intervals.

The level of agreement among the radiologist, gynecologist, and AI system was quantified using Cohen’s kappa (κ) coefficient and its 95% confidence interval. Because kappa statistics may be influenced by prevalence and marginal distribution imbalances, Gwet’s agreement coefficient (AC1) was also calculated as a complementary and more robust measure of concordance. The magnitude of agreement was interpreted according to the classification proposed by Landis and Koch.

AI was used to assist in the preparation of graphical visualizations based on statistical outputs generated by SPSS. The statistical analyses and results were produced exclusively using SPSS, while the AI tool was used only to facilitate the presentation of these results. All AI-assisted visualizations were reviewed and approved by the authors.

All analyses were based on two-tailed statistical tests, and statistical significance was defined as a *p* value below 0.05.

## 3. Results

A total of 1443 HSG images obtained from 414 women undergoing infertility evaluation were independently assessed by a radiologist, a gynecologist, and an AI system. The mean age of the study population was 30.97 ± 5.59 years (range, 19–45 years), and the average duration of infertility was 1.88 ± 0.61 years (Table 1).

Primary infertility was present in 364 women (87.9%), whereas 50 women (12.1%) had secondary infertility. The mean number of images acquired per examination was 3.49 ± 1.05, with a median of 4 (range, 1–8). Local anesthesia was used in 216 patients (52.2%), while 198 patients (47.8%) underwent the procedure under general anesthesia (Table 1).

Agreement analyses based on Cohen’s kappa statistics demonstrated the highest concordance for image artifact detection (κ = 0.726–0.799), followed by assessment of contrast medium distribution. For contrast distribution, substantial agreement was observed between the AI system and the radiologist (κ = 0.757), as well as between the AI system and the gynecologist (κ = 0.717).

Assessment of tubal patency showed moderate agreement between the radiologist and the gynecologist for both the right tube (κ = 0.536) and the left tube (κ = 0.453). In contrast, agreement between the AI system and the human evaluators was lower for tubal patency assessment. For peritoneal spill evaluation, agreement ranged from moderate to substantial across all comparisons, with the highest concordance observed between the AI system and the gynecologist (κ = 0.654).

Regarding overall HSG interpretation, substantial agreement was identified between the AI system and the radiologist (κ = 0.637, 95% CI: 0.563–0.711), exceeding the agreement observed between the radiologist and the gynecologist (κ = 0.363). Lower kappa coefficients were obtained for the evaluation of uterine filling defects, uterine anomalies, and tubal patency. Notably, agreement between the AI system and the radiologist was poor for right tubal patency (κ = 0.098). Detailed agreement coefficients are presented in Table 2.

**Figure 1:**
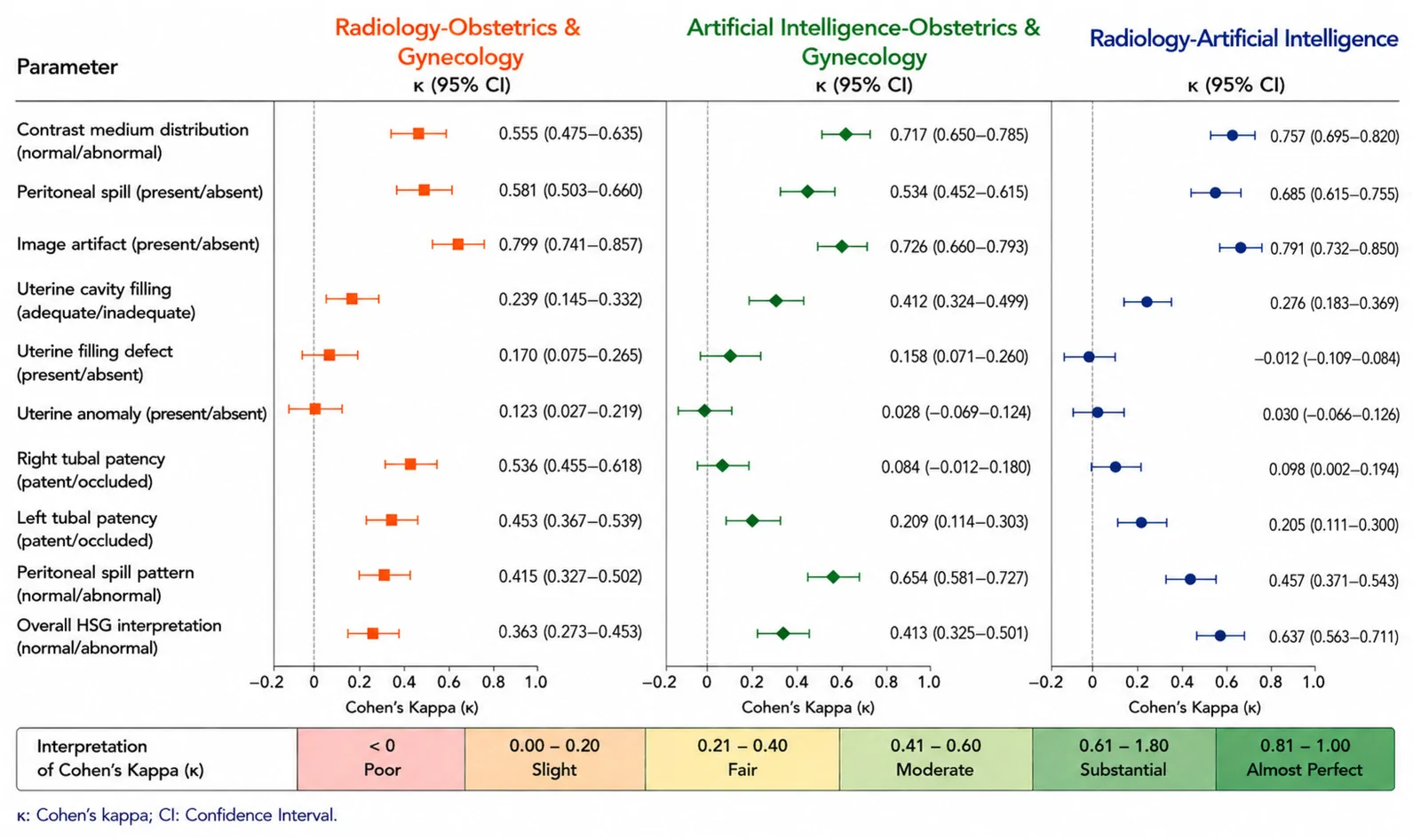
Interobserver agreement between Radiology, Obstetrics & Gynecology, and Artificial Intelligence in hysterosalpingography interpretation. Note: Forest plots illustrate Cohen’s kappa (κ) coefficients with 95% confidence intervals (CI) for pairwise agreement be-tween Radiology and Artificial Intelligence (blue), Radiology and Obstetrics & Gynecology (orange), and Artificial Intelligence and Obstetrics & Gynecology (green) across individual hysterosalpingography parameters. Agreement levels were interpreted according to standard Cohen’s kappa categories: poor (<0), slight (0.00–0.20), fair (0.21–0.40), moderate (0.41–0.60), substantial (0.61–0.80), and almost perfect (0.81–1.00). The highest agreement was observed for artifact detection and contrast medium assessment, whereas uterine filling defects and uterine anomalies demonstrated poor to slight agreement. Overall agreement was substantial between Radiology and Artificial Intelligence, moderate between Artificial Intelligence and Obstetrics & Gynecology, and fair to moderate between Radiology and Obstetrics & Gynecology.

**Figure 2:**
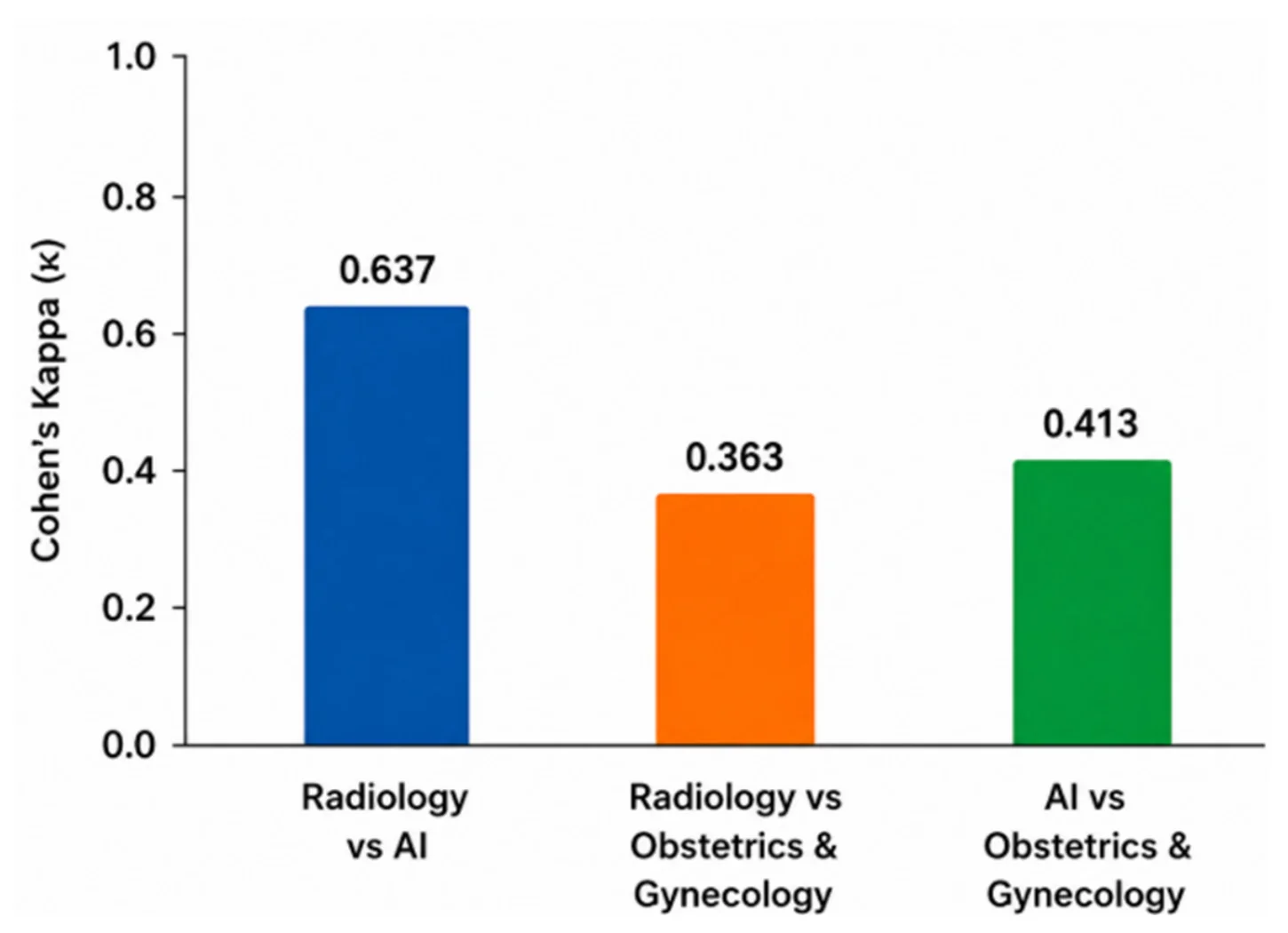
Overall agreement between observers. **Note:** Overall Cohen’s kappa (κ) values for pairwise comparisons in hysterosalpingography interpretation. Agreement was highest between Radiology and Artificial Intelligence (κ = 0.637), followed by Artificial Intelligence and Obstetrics & Gynecology (κ = 0.413), and Radiology and Obstetrics & Gynecology (κ = 0.363).

Interobserver agreement across diagnostic categories is illustrated in Figure 1. Most imaging parameters demonstrated moderate to substantial agreement, whereas lower levels of concordance were observed for selected uterine and tubal findings. The overall agreement patterns among evaluators are summarized in Figure 2.

Agreement estimates derived from Gwet’s AC1 were consistently higher than the corresponding kappa coefficients across nearly all evaluated parameters. Between the AI system and the radiologist, agreement was near perfect for contrast medium distribution (AC1 = 0.954, 95% CI: 0.930–0.972), peritoneal spill (AC1 = 0.893, 95% CI: 0.854–0.932), and peritoneal spill pattern (AC1 = 0.841, 95% CI: 0.787–0.878).

Similarly, agreement for overall HSG interpretation remained high between the AI system and the radiologist (AC1 = 0.700, 95% CI: 0.642–0.756). Detailed AC1 results are presented in Table 3.

The number of images obtained per patient did not differ according to infertility type (*p* = 0.314). However, examinations performed under local anesthesia yielded significantly more images than those performed under general anesthesia (3.86 ± 0.86 vs. 3.08 ± 1.10, *p* < 0.001) (Table 4).

Univariable logistic regression analyses demonstrated no significant association between secondary infertility and tubal pathology (OR = 1.34, 95% CI: 0.72–2.51, *p* = 0.358). Likewise, the anesthetic technique was not significantly associated with the presence of abnormal HSG findings (OR = 1.29, 95% CI: 0.88–1.90, *p* = 0.196) (Table 5).

In multivariable linear regression analysis, the use of general anesthesia was independently associated with a lower number of acquired images (β = −0.783, 95% CI: −0.988 to −0.577, *p* < 0.001). Conversely, the presence of tubal pathology was independently associated with a greater number of images obtained during the examination (β = 0.289, 95% CI: 0.030–0.547, *p* = 0.029).

No independent associations were identified between image count and patient age, duration of infertility, infertility type, or the presence of overall abnormal HSG findings (Table 6).

## 4. Discussion

In the present study, interobserver agreement among a radiologist, a gynecologist, and a multimodal artificial intelligence system was evaluated in the interpretation of hysterosalpingography examinations performed for infertility assessment. The findings demonstrated that the AI system achieved substantial agreement with expert evaluators, particularly for parameters requiring predominantly technical image interpretation. One of the most notable observations was that agreement between the AI system and the radiologist for overall HSG interpretation exceeded the level of agreement observed between the radiologist and the gynecologist. Importantly, the objective of this study was not to develop or validate an AI model specifically designed for HSG interpretation. Rather, we sought to explore the capability of a commercially available, general-purpose multimodal large language model to evaluate HSG images. Consequently, the results should be interpreted as reflecting the degree of concordance between AI-generated and expert assessments, providing insight into the consistency of AI-assisted HSG interpretation relative to expert evaluation.

The interpretation of HSG images is often subject to interobserver variability, a longstanding challenge that may affect diagnostic consistency in reproductive imaging. Beyond the assessment of static anatomical structures, HSG requires evaluation of the dynamic passage of contrast medium through the uterine cavity, fallopian tubes, and peritoneal cavity, making image interpretation inherently complex. Consequently, discrepancies among evaluators are not unexpected, even when identical image sets are reviewed. Consistent with this notion, Karadağ et al. reported significant variability between radiologists and gynecologists in the interpretation of HSG findings, particularly regarding tubal abnormalities and uterine cavity pathologies. Their findings suggest that differences in clinical background, training, and interpretative approaches may contribute to inconsistent assessments of the same examination [15]. Likewise, Glatstein et al. showed that discrepancies in the interpretation of HSG examinations can have important clinical implications, potentially affecting diagnostic conclusions as well as subsequent management strategies [16]. The moderate concordance between the radiologist and the gynecologist identified in the present study corroborates previous evidence demonstrating substantial interobserver variability in the interpretation of HSG examinations.

One of the most noteworthy findings of the present study was the relatively high level of agreement between the AI system and expert evaluators for technically oriented and relatively objective parameters, including image artifacts, contrast medium distribution, and peritoneal spill. The high kappa coefficients observed for image artifact detection suggest that the model was able to reliably identify technical factors affecting image quality. Similarly, the substantial agreement between the AI system and expert evaluators in the assessment of contrast medium distribution indicates that AI can consistently recognize fundamental visual features of HSG examinations. These findings suggest that multimodal AI systems may be particularly effective when evaluating imaging characteristics that rely primarily on pattern recognition and objective visual assessment. Supporting this interpretation, Yi et al. recently developed the HSG-Assistant system and demonstrated the feasibility of applying artificial intelligence to the automated analysis of HSG images. Their results further highlighted the potential for AI-assisted approaches to be integrated into routine HSG interpretation and clinical infertility practice [17]. Our findings further support these observations and suggest that AI systems are capable of generating interpretations that closely approximate those of expert radiologists, rather than merely performing basic image analysis. However, agreement between the AI system and human evaluators was notably lower for the assessment of tubal patency. Unlike the evaluation of static imaging features, determination of tubal patency requires interpretation of the dynamic progression of contrast medium through the fallopian tubes and into the peritoneal cavity. This process involves the integration of multiple imaging findings acquired over time and may therefore represent a more challenging task for general-purpose AI systems. Previous studies have similarly emphasized the technical limitations of imaging modalities and the critical role of interpreter expertise in the assessment of tubal patency. Consequently, variability in the evaluation of tubal passage remains one of the most challenging aspects of HSG interpretation, even among experienced clinicians [18,19]. This may partly explain why current AI models tend to exhibit lower performance when interpreting dynamic fluoroscopic processes compared with more static imaging features. In our study, particularly high levels of agreement were observed for the assessment of image artifacts and contrast medium distribution, suggesting that AI systems can reliably identify objective technical findings on HSG examinations. One such finding is contrast intravasation, a relatively common technical phenomenon during HSG that may lead to false-positive interpretations if not correctly recognized. Recent studies have suggested that intravasation should not be viewed solely as a procedural complication but also as a potentially meaningful radiologic finding associated with underlying tubal pathology [20,21]. The capacity of AI systems to consistently detect such technical findings may represent an important step toward improving the standardization, reproducibility, and objectivity of HSG interpretation.

A major limitation of current AI systems in clinical practice is their limited ability to incorporate broader clinical context. In infertility evaluation, HSG findings are typically interpreted alongside patient-specific factors such as age, infertility duration and type, history of pelvic inflammatory disease, and assisted reproductive treatments. In contrast, AI models based primarily on imaging data may not fully capture these clinical factors. Recent research has therefore focused on multimodal approaches that integrate imaging with electronic health records and other clinical data to provide more clinically relevant and personalized assessments [22,23]. Nelson et al. highlighted the potential value of combining biomedical datasets with electronic health records [22], while Kansagra et al. emphasized the growing importance of integrating imaging, large-scale datasets, and patient-specific clinical information in radiology informatics [23]. Previous studies of HSG have primarily focused on pain management and patient experience, with less attention to factors affecting image acquisition. Evidence suggests that NSAIDs combined with topical and paracervical lidocaine can provide effective analgesia during HSG [24,25]. In the present study, general anesthesia was independently associated with fewer acquired images, whereas tubal pathology was associated with a greater number of images. These findings suggest that both procedural factors and underlying HSG findings may influence image acquisition during the examination.

One of the notable methodological findings of the present study was the discrepancy observed between Cohen’s kappa and Gwet’s AC1 agreement coefficients. In particular, for low-prevalence findings such as uterine anomalies and intrauterine filling defects, kappa values remained relatively low despite AC1 coefficients indicating moderate to substantial agreement. This pattern suggests that the conventional kappa statistic may have been affected by the well-recognized prevalence paradox. Gwet demonstrated that Cohen’s κ statistic can underestimate agreement despite high observed concordance, whereas the AC1 coefficient provides more stable estimates by reducing the influence of prevalence-related bias [26]. Methodological studies have demonstrated that, in the presence of skewed category distributions, Gwet’s AC1 is less affected by prevalence and marginal probability effects than Cohen’s κ, resulting in a more robust and reliable measure of interobserver agreement [27]. Accordingly, the discrepancy between kappa and AC1 estimates observed in our study highlights the importance of employing complementary agreement metrics when evaluating low-frequency imaging findings. In the context of HSG, where several clinically relevant abnormalities may be relatively uncommon, exclusive reliance on Cohen’s κ may lead to an underestimation of observer agreement. The incorporation of Gwet’s AC1 may therefore provide a more balanced assessment of agreement across diagnostic categories with unequal prevalence. Nevertheless, very high AC1 values for rare abnormalities should be interpreted cautiously, as the predominance of concordant negative classifications may contribute to high agreement estimates. Therefore, high AC1 values in low-prevalence categories should not be interpreted as direct evidence of high diagnostic performance in detecting these specific abnormalities.

Although AI-assisted interpretation may contribute to greater standardization of HSG assessment by reducing observer-dependent variability, reproducibility remains an important consideration before the clinical implementation of novel imaging approaches. Previous studies evaluating emerging quantitative imaging techniques have emphasized the importance of formally assessing both intraobserver repeatability and interobserver reproducibility before their broader clinical application [28]. In the present study, agreement between the AI system and human evaluators was assessed; however, the repeatability of the AI system itself across repeated evaluations of the same HSG images was not directly investigated. Therefore, future studies should specifically evaluate the test–retest reproducibility of AI-generated HSG interpretations under standardized conditions.

Although the present findings suggest a potential role for AI-assisted HSG interpretation, its clinical utility remains to be established. Prospective studies incorporating independent reference standards are required to determine whether AI assistance can improve interpretation consistency, clinical decision-making, or workflow efficiency.

### Strengths and Limitations

The present study has several strengths. To the best of our knowledge, it is among the few studies to directly compare HSG interpretations by a radiologist, a gynecologist, and a multimodal AI system using the same image dataset. The inclusion of 1443 images from 414 HSG examinations provided a substantial dataset, and the use of both Cohen’s kappa and Gwet’s AC1 allowed a complementary assessment of interobserver agreement.

Several limitations should be considered. Agreement between the AI system and human evaluators was lower for clinically important findings such as tubal patency, uterine anomalies, and intrauterine filling defects. In addition, the AI system evaluated HSG images without access to clinical information, whereas HSG findings in routine practice are interpreted in conjunction with patient history and other clinical factors. The absence of this context may have affected AI-generated interpretations, particularly for more complex findings.

The AI system used in this study was a general-purpose multimodal large language model that had not been specifically trained, fine-tuned, or calibrated for HSG interpretation. Moreover, only one radiologist and one gynecologist participated; therefore, the observed agreement cannot be generalized to either professional group or to AI systems specifically developed for reproductive imaging.

No independent reference standard, such as laparoscopic or hysteroscopic confirmation, consensus adjudication, or documented reproductive outcomes, was available. Accordingly, this study should be regarded as an exploratory interobserver agreement study rather than a diagnostic accuracy study. Agreement reflects consistency between evaluators and does not establish diagnostic validity. Therefore, sensitivity, specificity, and other measures of diagnostic accuracy could not be determined, and the numerically higher agreement between the AI system and the radiologist should not be interpreted as evidence that either assessment was closer to the underlying diagnostic truth.

The inherently dynamic nature of HSG represents another important limitation. Because the AI evaluated sequential static images, it could not assess real-time contrast progression during fluoroscopy. This loss of temporal information may have particularly affected the assessment of tubal patency. The observed association between anesthetic technique and the number of acquired images may similarly reflect procedural dynamics. General anesthesia may allow a more standardized and uninterrupted examination with fewer acquisitions, whereas pain, suspected tubal spasm, or apparent obstruction in awake patients may prompt additional imaging. The independent association between tubal pathology and increased image number supports this interpretation; however, these mechanisms were not directly assessed and should be considered hypothesis-generating.

Operational reproducibility is also limited by the use of GPT-5 through the commercial ChatGPT web interface without access to a fixed model snapshot or API-versioned deployment. Because web-based AI platforms may undergo provider-side updates over time, the exact AI environment may not be fully reproducible, and identical inputs may not necessarily yield identical outputs in future versions.

The retrospective, single-center design further limits the generalizability of the findings. Differences in patient populations, imaging protocols, equipment, and clinical practice across institutions may affect observer agreement. Future prospective, multicenter studies incorporating multiple expert evaluators, independent clinical reference standards, and external validation across diverse populations are needed to confirm these findings and better define the potential role of AI-assisted HSG interpretation in clinical practice.

## 5. Conclusions

In conclusion, the AI system showed substantial agreement with expert evaluators for several HSG parameters, particularly contrast medium distribution, image artifacts, peritoneal spill, and peritoneal spill patterns. Agreement between the AI system and the radiologist for overall HSG interpretation was numerically higher than that between the radiologist and the gynecologist; however, lower agreement was observed for tubal patency and certain uterine findings. These results should be interpreted as measures of interobserver agreement rather than diagnostic accuracy, as no independent reference standard was available.

The retrospective single-center design, institution-specific HSG protocols, local anesthesia and image-acquisition practices, and the high proportion of patients with primary infertility may limit the generalizability of the findings. Therefore, the present results should not be considered evidence supporting immediate clinical implementation of AI-assisted HSG interpretation. Future prospective, multicenter studies involving diverse patient populations, multiple expert evaluators, independent clinical reference standards, and external validation are needed to determine the diagnostic validity and potential clinical role of AI-assisted HSG interpretation.

## Figures and Tables

**Table 1:** Baseline characteristics of the study population (n = 414).

Variable	Value
Age (years)	30.97 ± 5.59
Median age (range)	30 (19–45)
Infertility duration (years)	1.88 ± 0.61
Primary infertility, n (%)	364 (87.9%)
Secondary infertility, n (%)	50 (12.1%)
Median infertility duration (range)	2 (1–4)
Local anesthesia, n (%)	216 (52.2%)
General anesthesia, n (%)	198 (47.8%)
Number of HSG images per patient	3.49 ± 1.05
Median number of HSG images (range)	4 (1–8)
Total number of HSG images acquired	1443

Note: Data are presented as mean ± standard deviation, median (range), or number (%).

**Table 2:** Interobserver agreement among Radiology, Obstetrics & Gynecology and Artificial Intelligence for HSG interpretation.

Parameter	Radiology–Obstetrics & Gynecology κ (95% CI)	*p*-Value	Artificial Intelligence–Obstetrics & Gynecology κ (95% CI)	*p*-Value	Radiology–Artificial Intelligence κ (95% CI)	*p*-Value
**Contrast medium distribution (normal/abnormal)**	0.555 (0.475–0.635)	**<0.001**	0.717 (0.650–0.785)	**<0.001**	0.757 (0.695–0.820)	**<0.001**
**Peritoneal spill (present/absent)**	0.581 (0.503–0.660)	**<0.001**	0.534 (0.452–0.615)	**<0.001**	0.685 (0.615–0.755)	**<0.001**
**Image artifact (present/absent)**	0.799 (0.741–0.857)	**<0.001**	0.726 (0.660–0.793)	**<0.001**	0.791 (0.732–0.850)	**<0.001**
**Uterine cavity filling (adequate/inadequate)**	0.239 (0.145–0.332)	**<0.001**	0.412 (0.324–0.499)	**<0.001**	0.276 (0.183–0.369)	**<0.001**
**Uterine filling defect (present/absent)**	**0.170 (0.075–0.265)**	**<0.001**	**0.158 (0.071–0.260)**	**<0.001**	−0.012 (−0.109–0.084)	0.804
**Uterine anomaly (present/absent)**	**0.123 (0.027–0.219)**	**0.012**	**0.028 (−0.069–0.124)**	0.572	0.030 (−0.066–0.126)	0.542
**Right tubal patency (patent/occluded)**	0.536 (0.455–0.618)	**<0.001**	0.084 (−0.012–0.180)	0.086	0.098 (0.002–0.194)	**0.045**
**Left tubal patency (patent/occluded)**	0.453 (0.367–0.539)	**<0.001**	0.209 (0.114–0.303)	**<0.001**	0.205 (0.111–0.300)	**<0.001**
**Peritoneal spill pattern (normal/abnormal)**	0.415 (0.327–0.502)	**<0.001**	0.654 (0.581–0.727)	**<0.001**	0.457 (0.371–0.543)	**<0.001**
**Overall HSG interpretation (normal/abnormal)**	0.363 (0.273–0.453)	**<0.001**	0.413 (0.325–0.501)	**<0.001**	0.637 (0.563–0.711)	**<0.001**

**Note:** For comparisons with κ > 0.20, the observed agreement was statistically significantly greater than chance (*p* < 0.001).

**Table 3:** Gwet’s AC1 agreement coefficients for HSG interpretation.

Parameter	Radiology–Obstetric & Gynecology AC1 (95% CI)	Artificial Intelligence–Obstetric & Gynecology AC1 (95% CI)	Radiology–Artificial Intelligence AC1 (95% CI)
**Contrast medium distribution (normal/abnormal)**	0.917 (0.884–0.945)	0.958 (0.939–0.979)	0.954 (0.930–0.972)
**Peritoneal spill (present/absent)**	0.891 (0.845–0.922)	0.871 (0.810–0.902)	0.893 (0.854–0.932)
**Image artifact (present/absent)**	0.805 (0.747–0.856)	0.734 (0.660–0.790)	0.793 (0.731–0.849)
**Uterine cavity filling (adequate/inadequate)**	0.710 (0.643–0.778)	0.860 (0.817–0.900)	0.687 (0.616–0.754)
**Uterine filling defect (present/absent)**	0.690 (0.640–0.741)	0.856 (0.819–0.893)	0.602 (0.551–0.655)
**Uterine anomaly (present/absent)**	0.780 (0.732–0.814)	0.797 (0.754–0.832)	0.659 (0.610–0.701)
**Right tubal patency (patent/occluded)**	0.845 (0.800–0.878)	0.747 (0.683–0.802)	0.725 (0.665–0.778)
**Left tubal patency (patent/occluded)**	0.830 (0.787–0.873)	0.777 (0.730–0.821)	0.751 (0.704–0.803)
**Peritoneal spill pattern (normal/abnormal)**	0.860 (0.812–0.898)	0.944 (0.920–0.967)	0.841 (0.787–0.878)
**Overall HSG interpretation (normal/abnormal)**	0.418 (0.354–0.481)	0.465 (0.400–0.532)	0.700 (0.642–0.756)

**Note:** Interobserver agreement was additionally assessed using Gwet’s Agreement Coefficient (AC1). AC1 was reported to reduce the influence of prevalence and marginal probability effects that may affect Cohen’s κ estimates. No bootstrap resampling was performed.

**Table 4:** Number of HSG images obtained per patient according to infertility type and anesthesia method.

Variable	n	Mean ± SD	Median (Min–Max)	*p*-Value
**Infertility type**				
**Primary infertility**	364	3.47 ± 1.09	4 (1–8)	0.314
**Secondary infertility**	50	3.58 ± 0.76	4 (2–6)	
**Anesthesia method**				
**Local anesthesia**	216	3.86 ± 0.86	4 (1–8)	**<0.001**
**General anesthesia**	198	3.08 ± 1.10	3 (1–8)	

**Note:** Continuous variables are presented as mean ± standard deviation and median (minimum–maximum). Comparisons were performed using the Mann–Whitney U test (or Student’s *t*-test if normally distributed).

**Table 5:** Association of infertility type and anesthesia method with clinical outcomes.

Variable	Group	Event n (%)	No Event n (%)	OR (95% CI)	*p*-Value
**Tubal pathology**	**Primary infertility (n = 364)**	101 (27.7)	263 (72.3)	Reference	0.358
	**Secondary infertility (n = 50)**	17 (34.0)	33 (66.0)	1.34 (0.72–2.51)	
**Abnormal HSG findings**	**Local anesthesia (n = 216)**	103 (47.7)	113 (52.3)	Reference	0.196
	**General anesthesia (n = 198)**	107 (54)	91 (46)	1.29 (0.88–1.90)	

**Note:** The effects of infertility type and anesthesia method on clinical outcomes were evaluated using binary logistic regression analysis. Odds ratios (ORs) and their corresponding 95% confidence intervals (CIs) were calculated. Primary infertility and local anesthesia were used as the reference categories. Comparisons of categorical variables between groups were performed using the chi-square (χ^2^) test.

**Table 6:** Multivariable linear regression analysis for factors associated with the number of HSG images obtained per patient. HSG, hysterosalpingography; CI, confidence interval.

Variable	β Coefficient (95% CI)	*p* Value
**Age (per year)**	0.033 (−0.004 to 0.071)	0.082
**Infertility duration (per year)**	−0.285 (−0.595 to 0.025)	0.072
**Secondary vs. primary infertility**	−0.212 (−0.646 to 0.223)	0.339
**General vs. local anesthesia**	−0.783 (−0.988 to −0.577)	**<0.001**
**Pathological vs. normal HSG**	0.146 (−0.088 to 0.380)	0.221
**Tubal pathology present vs. absent**	0.289 (0.030 to 0.547)	0.029

**Note:** β coefficients represent the adjusted change in the number of HSG images obtained per patient after controlling for all variables included in the model.

## Data Availability

The datasets generated and/or analyzed during the current study are available from the corresponding author upon reasonable request.
